# Autologous Platelet Rich Plasma As A Preparative for Resurfacing Burn Wounds with Split Thickness Skin Grafts

**DOI:** 10.29252/wjps.9.1.29

**Published:** 2020-01

**Authors:** Samarth Gupta, Pradeep Goil, Sangeeta Thakurani

**Affiliations:** Department of Plastic, Reconstructive and Burns, SMS Hospital Jaipur, Jaipur, Rajasthan, India

**Keywords:** Autologous, Platelet rich plasma, Burn, Wound, Skin graft

## Abstract

**BACKGROUND:**

Split thickness skin graft is a widely accepted technique to cover large defects. Shearing, hematoma and infection have often been attributed as major causes for graft loss. Autologous platelet rich plasma (PRP) has been used in various treatment modalities in the field of plastic surgery for its healing, adhesive and hemostatic properties owing to the growth factors that are released. This Study primarily throws light on the usage of PRP over difficult Burn wound beds to augment graft uptake and attenuate complications.

**METHODS:**

The patients were divided into two groups of those who were subjected to use of autologous PRP as a preparative burn surfacing and the control group who underwent standard method of treatment.

**RESULTS:**

Patients in PRP group significantly showed a higher graft adherence rate as compared to those with other method. It also reduced pain, and hematoma formation.

**CONCLUSION:**

Application of PRP is a safe, cost effective, easy method to increase graft adherence rate in patients with burns where graft loss is noticed and there is shortage of donor sites.

## INTRODUCTION

Split thickness skin graft is a widely accepted technique to cover large defects. Shearing, hematoma and infection have often been attributed as major causes for graft loss. Autologous platelet rich plasma (PRP) has been used in various treatment modalities,^1^^-^^4^ including the field of plastic surgery for its healing, adhesive and hemostatic properties owing to the growth factors that are released.^5^^-^^8^

Platelet-rich plasma (PRP) is an autologous product that concentrates a large number of platelets in a small volume of plasma.^9^^,^^10^ PRP functions as a fibrin tissue adhesive with hemostatic and tissue sealing properties, but it differs from fibrin glue and other platelet-poor tissue adhesives because its platelets provide a unique ability to promote wound healing.^11^ PRP provides an immediate surgical hemostatic agent that is biocompatible, safe, and effective. It accelerates endothelial, epithelial, and epidermal regeneration, stimulates angiogenesis, enhances collagen synthesis, promotes soft tissue healing, decreases dermal scarring, enhances the hemostatic response to injury, and the high leukocyte concentration of PRP has an added antimicrobial effect.^12^ The application of autologous PRP to STSG sites has been recently described for immediate skin graft anchorage as well as inosculation of the STSG with nutrient-rich blood media.^13^^,^^14^ This Study primarily throws light on the usage of PRP over difficult burn wound beds to augment graft uptake and attenuate complications. 

## MATERIALS AND METHODS

The study was performed in Department of Plastic, Reconstructive and Burns Surgery, SMS Hospital, Jaipur, India between 2017 and 2019. Totally, 200 patients were enrolled with written and informed consent and were divided into two groups of 100 each as case and control on a randomized basis. The patients were initially admitted and were resuscitated. They underwent dressings until the bed was ready for grafting. The patients in the cases group were subjected to use of PRP just before application of skin grafts. The patients in the control group underwent grafting by the standard method.

The inclusion criteria were all patients with burns and healing ulcers and a serum albumin above 2.5 g/dL. The exclusion criteria were an active infection, age >70 years, presence of pus and discharge, immune-compromised patients, exposed ligaments/bone/cartilage, underlying comorbidities, uncooperative patients and drug abuse. To prepare PRP based on various reported methods,^13^^ p^atients venous sample was taken, collected in 10 mL ACD vials, and spinned for 10 min at 3500 rpm. PRP was applied on the wound bed as a thin film just before applying the graft and 5 mL for 100 square cm was used. Additional sutures were taken to fix as necessary. Non adhesive paraffin gauze dressing was applied over the grafted area. Dressing was conducted on day 2^nd^ and subsequently on alternate days. Graft adherence was investigated on day 2^nd^ and day 6^th^. Any hematoma formation and the cost were evaluated.

## RESULTS

There were 59 and 48 cases and controls for males, respectively and these figures for females were 41 and 52, respectively. The mean age of patients was 45.1 years in case and 48.2 years in control group. Graft adherence on day 2^nd^ was 89 and 76 on day 6^th^ for case and control groups, respectively. These figures on day 2^nd^ and 6^th^ in case and control groups were 85 and 70, respectively. All 100 patients who received the intervention (88.86±34.5) demonstrated significantly better graft uptake rate on day 2^nd^ [t(51)=2.1 *p*=0.04], when compared to the 100 patients in the control group (425±31). 

There was no significant difference for sex, [t(8)=1.7, *p*=0.097], even women (55±8) attained higher score than men (53±7.8). The overall cost of PRP was only 200-300 Rupees, while staples and sutures’ cost was 2000-3000 Rupees. Two patients in the case group showed hematoma formation; while in the control group, hematoma formation was noticed in 8 patients. About 50% graft loss was observed in the case group (both of them were in the region of neck and axilla), while in the control group, 6 patients developed signs of local infection with 70%, 40%, 50%, 50%, 50%, and 70% graft loss. Donor site healing was uneventful and had a good aesthetic outcome in both the groups.

## DISCUSSION

PRP is a biological product defined as a portion of the plasma fraction of autologous blood with a platelet concentration above the baseline. It is obtained from the blood of patients and collected before centrifugation.^12^^,^^15^ It helps early vascularization by delivering these growth factors and increasing collagen synthesis. There are other benefits like reduced hematoma, infection, and cost. It has three stages of anchorage, inosculation and maturation, while the success of the first two stages is critical to the overall success of serum imbibition; revascularization (anastomosis, neovascularization, and endothelial cell ingrowth); and maturation. 

In STSGs, keratinocytes on the basal layer show high proliferation rates, which may ultimately stimulate growth factor excretion. Chronic wounds may lack growth factors due to decreased production and release, trapping, excess degradation, or a combination of these mechanisms; thus delaying wound healing, which is overcome by PRP. While fibrin is crucial for early cell migration in the wound bed, collagen deposition allows wound maturation.^16^ Normally, the platelet concentration is described as low: 0.5x, intermediate: 0.5-3, and high: 3-5 million/µl.

We have been able to achieve high concentration of platelets by our technique of preparation. Use of PRP has been studied by many researchers. According to Gibran *et al.*’s study done on forty post burn patients, PRP was safe and effective for fixation of skin grafts due to its adhesive nature and outcomes were better than securing skin graft to wound margins or bed with sutures, staples or glue.^17^ Schade and Roukis found that addition of PRP to split-thickness skin graft recipient sites enhanced primary healing and reduced healing time, likely as a result of shearing force reduction and enhancement of the wound environment with growth factors.^18^ A study by Kakudo *et al.* revealed that PRP promoted epithelialization and angiogenesis of split-thickness skin graft donor sites.^19^ In a recent study, Wanden-Berghe *et al.* found clinically a clear improvement in chronic wounds with an accelerated healing on application of PRP activated by calcium chloride.^20^ Application of PRP was shown to be safe, cost effective, easy to increase graft adherence in patients with burns, where graft losses are often seen and where there is a shortage of donor sites.

## CONFLICT OF INTEREST

The authors declare no conflict of interest.
